# Comparing researcher-curated AI-drafted feedback styles for negative automatic thought believability: an online randomized scenario-based experiment

**DOI:** 10.3389/fpsyg.2026.1926092

**Published:** 2026-10-01

**Authors:** Yu Liu, Junting Mu, Ying Li, Yingzi Ke, Yingge Zhang

**Affiliations:** 1 Faculty of Health and Wellness, City University of Macau, Macau, China; 2 Xianyang Normal University, Xianyang, Shaanxi, China; 3 Nanjing Taiheyu Health Management Co., Ltd., Nanjing, Jiangsu, China; 4 School of Management, Changsha Medical University, Changsha, Hunan, China

**Keywords:** AI-drafted feedback, cognitive reappraisal, cognitive restructuring, empathy, negative automatic thoughts, researcher-curated stimuli, Socratic questioning

## Abstract

**Introduction:**

AI-drafted feedback is increasingly used in learning support, emotional support, peer support, and self-guided reflection, yet empirical studies often combine emotional validation, advice, reappraisal, and Socratic questioning within the same response. This study compared four researcher-curated AI-drafted feedback styles: neutral information, empathetic feedback, Socratic challenge, and integrated feedback.

**Methods:**

An online randomized scenario-based experiment was conducted using four daily stress scenarios crossed with the four feedback conditions. After screening 427 questionnaire records collected from March 12 to March 20, 2026, valid responses from 389 adults were analyzed. Participants read one scenario, rated negative automatic thought (NAT) believability and negative affect, received one feedback text, completed post-test and feedback evaluation measures, and wrote a balanced alternative thought. Regression models controlled for pre-test scores, scenario, trait NAT tendency, AI trust, and trait reactance.

**Results:**

All three active feedback styles predicted lower post-test NAT believability than neutral information, with the largest adjusted difference observed for integrated feedback, followed by Socratic challenge and empathetic feedback. Empathetic feedback produced higher perceived empathy and greater affect reduction, whereas Socratic challenge produced higher cognitive reappraisal and reframing quality. Integrated feedback combined high feedback acceptance with strong evidence-based examination of the negative thought.

**Discussion:**

In this scenario-based task, researcher-curated AI-drafted feedback produced larger reductions in NAT believability and higher evidence-based reframing quality when emotional validation was followed by evidence testing, alternative explanation, and fact-inference distinction.

## 1 Introduction

Written feedback often helps people interpret setbacks, revisit negative judgments, and regulate emotional reactions in learning support, peer support, self-guided reflection, and emotional support settings (Fitzpatrick et al., 2017; Inkster et al., 2018; Vaidyam et al., 2019; Graham et al., 2019; Haque and Rubya, 2023). Generative AI now produces such feedback quickly, and its growing use makes it possible to compare different response styles under controlled conditions.

Yet AI responses often mix emotional validation, advice, reappraisal, and Socratic questioning in the same message, so it is difficult to tell which component is linked to belief change, affect reduction, or reframing (Malgaroli et al., 2023; Li et al., 2023; Stade et al., 2024). To compare feedback styles under controlled conditions, each response was first drafted with generative AI and then reviewed and fixed by the research team before it was presented to participants.

Negative automatic thoughts are rapid appraisals that arise in stressful situations and often contain negative self-evaluation, overgeneralized inference, or catastrophic interpretation. Cognitive theory treats these thoughts as interpretations through which events acquire emotional meaning (Beck et al., 1979; Beck, 2011). The Automatic Thoughts Questionnaire provided an early measurement foundation for negative self-statements (Hollon and Kendall, 1980), and later work distinguishes thought frequency from thought believability (Netemeyer et al., 2002; Batmaz and Ozdel, 2015). Accordingly, post-test NAT believability served as the immediate cognitive outcome after a single feedback exposure to a scenario-specific negative thought.

Cognitive restructuring asks a person to identify, test, and revise interpretations that are weakly supported or overly negative. Cognitive reappraisal, a related emotion-regulation strategy, changes the meaning assigned to an event and has shown reliable associations with emotional experience across experimental and individual-difference research (Gross, 1998; Gross and John, 2003; Webb et al., 2012; McRae and Gross, 2020). In an AI feedback task, reappraisal depends both on what the feedback asks the participant to do and on whether the participant is willing to take up the message. Empathetic feedback may lower defensive reception by acknowledging emotion, normalizing the reaction, and conveying respect (Rogers, 1957; Elliott et al., 2011; Norcross and Lambert, 2019). Socratic challenge may lower NAT believability by prompting evidence testing, fact-inference distinction, probability estimation, and alternative explanation generation (Clark and Egan, 2015; Braun et al., 2015).

Recent AI research has shown that large language models can generate replies that readers perceive as emotionally supportive or empathic, while related work has begun to test LLMs in cognitive restructuring, cognitive reappraisal, CBT-style tasks, and Socratic dialogue (Sharma et al., 2023; Ayers et al., 2023; Lee et al., 2024; Gabriel et al., 2024; Sharma et al., 2024; Zhan et al., 2024; Held et al., 2024, 2025; Hodson and Williamson, 2024; Xiao et al., 2024). These strands raise a sequencing question for integrated feedback. Emotional validation may make the feedback easier to receive; evidence testing may then help participants treat the negative thought as an interpretation that can be examined.

The online randomized scenario-based experiment compared neutral information, empathetic feedback, Socratic challenge, and integrated feedback. Four hypotheses were tested. H1 predicted that empathetic, Socratic, and integrated feedback would reduce NAT believability more than neutral information. H2 predicted that empathetic feedback would produce higher perceived empathy and stronger affect reduction than Socratic challenge. H3 predicted that Socratic challenge would produce higher cognitive reappraisal and reframing quality than empathetic feedback. H4 predicted that integrated feedback would produce the largest overall reduction in NAT believability while maintaining high feedback acceptance and cognitive reappraisal, with emotional validation preceding evidence-based examination of the negative thought. The experiment tests how feedback style affects NAT believability, negative affect, feedback acceptance, feedback reactance, cognitive reappraisal, and open-ended reframing quality.

## 2 Related work

### 2.1 Negative automatic thoughts and cognitive restructuring

Negative automatic thoughts are cognitive appraisals through which people interpret stressful events, personal ability, and social evaluation. They are closely tied to emotion, but their theoretical role is the interpretation that gives an event personal meaning. Beck's cognitive theory links automatic thoughts to emotional reactions and coping behavior, while the Automatic Thoughts Questionnaire treats negative self-statements as measurable cognitive events (Beck et al., 1979; Hollon and Kendall, 1980; Beck, 2011). This tradition supports a scenario-based task in which participants evaluate the credibility of one target thought after a specified stressor.

Measurement work helps distinguish the primary outcome from other ways of describing negative thoughts. Frequency measures indicate how often negative thoughts occur, whereas believability measures indicate whether a person treats a specific thought as true or convincing. In a short feedback experiment, believability provides a more sensitive marker of immediate cognitive flexibility than symptom scales or retrospective frequency estimates (Netemeyer et al., 2002; Batmaz and Ozdel, 2015). The feedback task therefore asks whether the original negative interpretation becomes less credible after participants read a fixed response.

Cognitive restructuring and cognitive reappraisal provide the process vocabulary for this outcome. Cognitive restructuring is rooted in CBT and emphasizes testing unsupported interpretations; cognitive reappraisal is rooted in emotion-regulation research and emphasizes changing the meaning of a situation (Gross, 1998; Gross and John, 2003; Webb et al., 2012; McRae and Gross, 2020). The two converge in the alternative explanations participants generate, the distinction they make between facts and inferences, and the lower believability they assign to the original thought.

### 2.2 Empathy, acceptance, and feedback reception

Empathy can influence cognitive change by shaping feedback reception. Supportive communication and psychotherapy research show that being understood, having emotion acknowledged, and experiencing acceptance can make difficult feedback easier to consider (Rogers, 1957; Elliott et al., 2011; Norcross and Lambert, 2019). In a brief AI-drafted text, perceived empathy is the participant's evaluation of the response: whether the text sounds understanding, respectful, and emotionally validating.

The wording matters. AI-generated responses can be perceived as empathic without implying that an AI system has human empathy. Human-AI collaboration studies show that AI can help increase empathic expression in peer support, and several studies report high reader ratings for some AI-generated supportive responses (Sharma et al., 2023; Ayers et al., 2023; Lee et al., 2024; Gabriel et al., 2024). Perceived empathy is therefore measured as a process indicator, with cognitive restructuring treated separately.

Empathetic feedback may be useful when a participant is emotionally activated and likely to resist advice. It can reduce the sense of being judged and increase willingness to consider the feedback. Accordingly, the analysis includes perceived empathy, feedback acceptance, feedback reactance, affect reduction, and evidence-based reframing as distinct outcomes.

### 2.3 Socratic questioning and cognitive challenge

Socratic questioning is a structured method for evidence testing. In CBT, Socratic dialogue helps individuals examine evidence, identify assumptions, estimate probabilities, and generate alternative interpretations (Clark and Egan, 2015). Empirical work also suggests that therapist use of Socratic questioning is associated with changes in cognition and symptoms across sessions (Braun et al., 2015). These functions guided the Socratic challenge condition, which prompted participants to test the evidential basis of the target thought.

AI-supported Socratic dialogue has become a specific research target. Studies have examined self-guided cognitive restructuring with language models, LLM-generated cognitive reappraisal responses, and CBT-style assistance (Sharma et al., 2024; Zhan et al., 2024; Hodson and Williamson, 2024). Socrates 2.0 was developed to guide users through Socratic dialogue around unhelpful beliefs, and psychotherapy-oriented model evaluations highlight the need to assess both language quality and task fit (Held et al., 2024, 2025; Xiao et al., 2024). Other mental-health language-model studies illustrate the broader growth of LLM methods for psychological text and support tasks (Xu et al., 2024; Yang et al., 2024).

For AI-drafted feedback, cognitive challenge must be phrased as an invitation to inspect a thought. A text that directly corrects the participant may increase feedback reactance; a text that asks about evidence, alternative explanations, and probabilities may help the participant treat the thought as testable. The Socratic challenge condition therefore used evidence-testing language, and feedback reactance was measured as perceived pressure, preachiness, and disrespect.

### 2.4 AI-generated mental health support and evaluation

Digital mental health and support tools have long promised low-threshold access, flexible timing, and scalable interaction, but their evidence bases differ across tool types and use cases (Fitzpatrick et al., 2017; Inkster et al., 2018; Vaidyam et al., 2019; Graham et al., 2019; Li et al., 2023; Haque and Rubya, 2023). Recent LLM reviews extend this discussion to psychoeducation, journaling support, screening assistance, and reflective feedback (Omar et al., 2024; Guo et al., 2024; Hua et al., 2025; Jin et al., 2025). This comparison fits the reflective feedback category by examining brief text responses to ordinary academic and social stress scenarios.

Evaluation becomes more precise when the response text is controlled before data collection. Researcher-curated AI-drafted texts allow a randomized comparison of response styles while keeping scenario content, task order, and outcome measurement constant. Recent reviews of AI mental health support also emphasize defined task scope, privacy protection, crisis-safety procedures, and clear accountability when AI-generated responses enter psychological support contexts (Lawrence et al., 2024; Bucher et al., 2025; Torous and Topol, 2025; Malgaroli et al., 2025; Steenstra and Bickmore, 2025; Rousmaniere et al., 2026). These concerns informed the stimulus-review and survey procedures for the feedback-style comparison.

## 3 Theoretical framework

### 3.1 Negative automatic thought believability as a short-term cognitive outcome

NAT believability captures how strongly a person accepts a situation-specific negative interpretation as true, plausible, and compelling. In a scenario-based feedback task, it offers a proximal cognitive outcome because the response addresses a particular thought after a particular stressor, rather than broader symptom severity or the general frequency of negative thoughts. The pre-test rating records the initial credibility of the target thought, whereas the post-test rating records how convincing that thought remains after exposure to the fixed feedback text. The measure stays close to the point at which feedback may loosen a specific interpretation.

The four conditions differ in the route by which they may affect that judgment. Neutral information offers a general frame for reflection without directly validating emotion or testing evidence. Empathetic feedback may make reconsideration easier by acknowledging the distress attached to the thought; Socratic challenge addresses the thought's evidential basis; and integrated feedback places validation before challenge. NAT believability is therefore the central cognitive comparison, while its change may also depend on whether participants experience the feedback as usable and respectful enough to engage with.

### 3.2 Affective reception and evidence-based examination

Emotional validation shapes the first point of contact between the participant and the response. A text that acknowledges distress, conveys respect, and avoids an immediately corrective tone is easier to remain engaged with when the target thought is tied to disappointment, uncertainty, or self-doubt. A negative interpretation is harder to examine when the response itself feels dismissive or judging. Recognition of the emotional reaction can leave the participant more willing to consider what the thought is based on and whether it is the only plausible reading of the event.

Evidence-based examination then directs attention to the thought's support and limits. Socratic challenge asks participants to separate what is known from what is inferred, consider alternative explanations, and judge how much weight the available evidence can carry. Integrated feedback begins with validation and moves into these questions, placing the request for reconsideration within a response that first acknowledges the participant's reaction. Validation concerns whether the message can be received; evidence testing concerns how the original interpretation is reconsidered once the message has been taken up. These two aspects may appear differently in NAT believability and negative affect, so the participant's immediate response to the feedback is part of the theoretical account.

### 3.3 Feedback acceptance, reactance, and reframing quality as process indicators

The way a response is received can be observed in whether participants regard it as useful and worth considering or experience it as preachy, pressuring, disrespectful, or overly challenging. Feedback acceptance and feedback reactance matter because the same request to examine evidence may be taken up as an invitation to reflect or resisted as an attempt to impose a conclusion. These reactions help locate differences in NAT believability and negative affect within the immediate experience of reading the response.

Open-ended reframing quality captures what participants put into their own words after reading the feedback. Higher scores require a distinction between facts and inferences, an alternative explanation, and a balanced conclusion, so the measure records a more concrete form of cognitive reworking than a self-report that the feedback was helpful. Acceptance, reactance, and reframing quality were all obtained after the same single feedback exposure and function as process indicators of that experience; they show how the response was received and the extent to which its style was reflected in the participant's own alternative thought.

## 4 Methods

### 4.1 Participants and data screening

Participants were recruited through the Wenjuanxing sample service and online university recruitment links. Data were collected from March 12, 2026 to March 20, 2026. Eligible participants had to be at least 18 years old, able to read Chinese daily stress scenario materials, and able to submit only one valid response. The survey platform recorded IP address, device identifier, and submission time for duplicate screening. When responses from the same device or IP address appeared within a short interval, only the first complete response was retained. The recruitment target was approximately 400 completed questionnaires; with the final valid sample of 389, a sensitivity analysis indicated 80% power to detect an omnibus four-group effect of *f* = 0.168 (η^2^ = 0.028) at α = 0.05.

The raw dataset contained 427 questionnaire records. Screening removed 38 records for too-fast completion, attention-check failure, incomplete core measures, or straight-line responding, leaving 389 valid responses for analysis. A response counted as too fast when its completion time fell below one-third of the full-sample median or below 180 seconds. Attention-check failure meant that a participant did not select the instructed option in an item such as “Please select ‘Agree' for this item.” Incomplete core measures covered missing pre-test NAT believability, post-test NAT believability, condition assignment, or scenario information. Straight-line responding was identified when the same response value was selected for at least 80% of items within a scale and the open-ended response contained fewer than five valid words.

The final sample included 211 women, 160 men, and 18 participants who selected another gender category or preferred not to disclose. Most participants were aged 18 to 25 years, and the sample was concentrated among undergraduate and graduate education stages. Valid group sizes were 94 in neutral information, 98 in empathetic feedback, 96 in Socratic challenge, and 101 in integrated feedback. Table 1 concentrates the recruitment window, eligibility rules, demographic composition, and exclusion counts, so the sample source and cleaning decisions can be read alongside one another. Figure 1 traces how the raw questionnaire file became the analytic dataset: 427 raw records were reduced to 389 valid responses after 38 exclusions, comprising too-fast completion (*n* = 12), attention-check failure (*n* = 10), incomplete core measures (*n* = 9), and straight-line responding (*n* = 7).

**Table 1:** Sample characteristics and screening summary.

Domain	Category	*N*
Recruitment and period	Wenjuanxing sample service; university recruitment links; March 12–20, 2026	–
Eligibility	Age 18 years or older; able to read Chinese scenario materials; no duplicate retained	–
Questionnaire records	Raw submitted records	427
Questionnaire records	Valid responses retained for analysis	389
Sample composition	Women	211
Sample composition	Men	160
Sample composition	Another gender or preferred not to disclose	18
Sample composition	Aged 18–25 years	308
Sample composition	Undergraduate or graduate students	330
Screening summary	Excluded responses	38
Screening summary	Too-fast completion	12
Screening summary	Attention check failed	10
Screening summary	Incomplete core measures	9
Screening summary	Straight-line responding	7
Screening summary	Duplicate submissions identified	0

**Figure 1:**
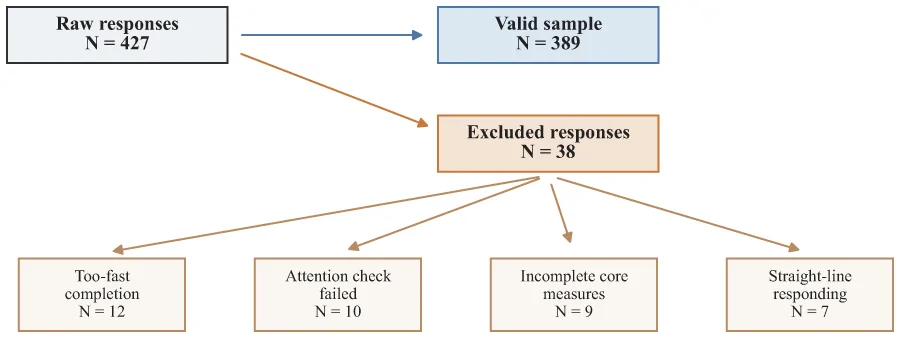
Response screening flow for raw, excluded, and valid responses.

### 4.2 Experimental design and random assignment

The experiment used a 4 × 4 between-subjects design crossing four daily stress scenarios with four feedback conditions. Scenario assignment and feedback condition assignment were independent random factors. Each participant read one scenario and received one feedback text.

The four scenarios represented common academic or social stressors, but they targeted different NAT triggers: academic ability doubt, ambiguous social rejection inference, competence-related self-evaluation after revision feedback, and public-evaluation concern after speaking hesitation. The mapping in Table 2 functions as a content check for the stimulus set by connecting each trigger to a target negative automatic thought and psychological focus. Because the scenarios differ in the type of negative interpretation they evoke, the experiment is less tied to a single stress domain or one narrow form of NAT.

**Table 2:** Daily stress scenarios and target negative automatic thoughts.

Scenario	Situational trigger	Target negative automatic thought	Psychological focus
Learning or classroom presentation failure	Poor learning outcome or unsuccessful classroom presentation	“I am not capable enough, and this failure shows I cannot do well.”	Ability doubt after academic setback
Delayed social response	Message remains unanswered longer than expected	“The other person may not care about me or may be rejecting me.”	Social-evaluation inference from ambiguous delay
Task revision feedback	A teacher or peer asks for substantial revision	“The feedback means my work is poor and I am not competent.”	Self-evaluation after corrective feedback
Public speaking hesitation	Participant hesitates or loses fluency while speaking publicly	“Others must think I performed badly.”	Catastrophic inference after visible hesitation

The reconstructed English scenario wording and target-thought translations are provided in Supplementary Table S3, which documents the structure and content of the experimental scenarios.

The valid cell counts for condition × scenario combinations ranged from 17 to 31. The randomization check in Figure 2 indicates that every condition-by-scenario combination remained analyzable, with no empty cells or extreme sparsity. Because the realized cell sizes were not identical, scenario was retained as a covariate in the primary models.

**Figure 2:**
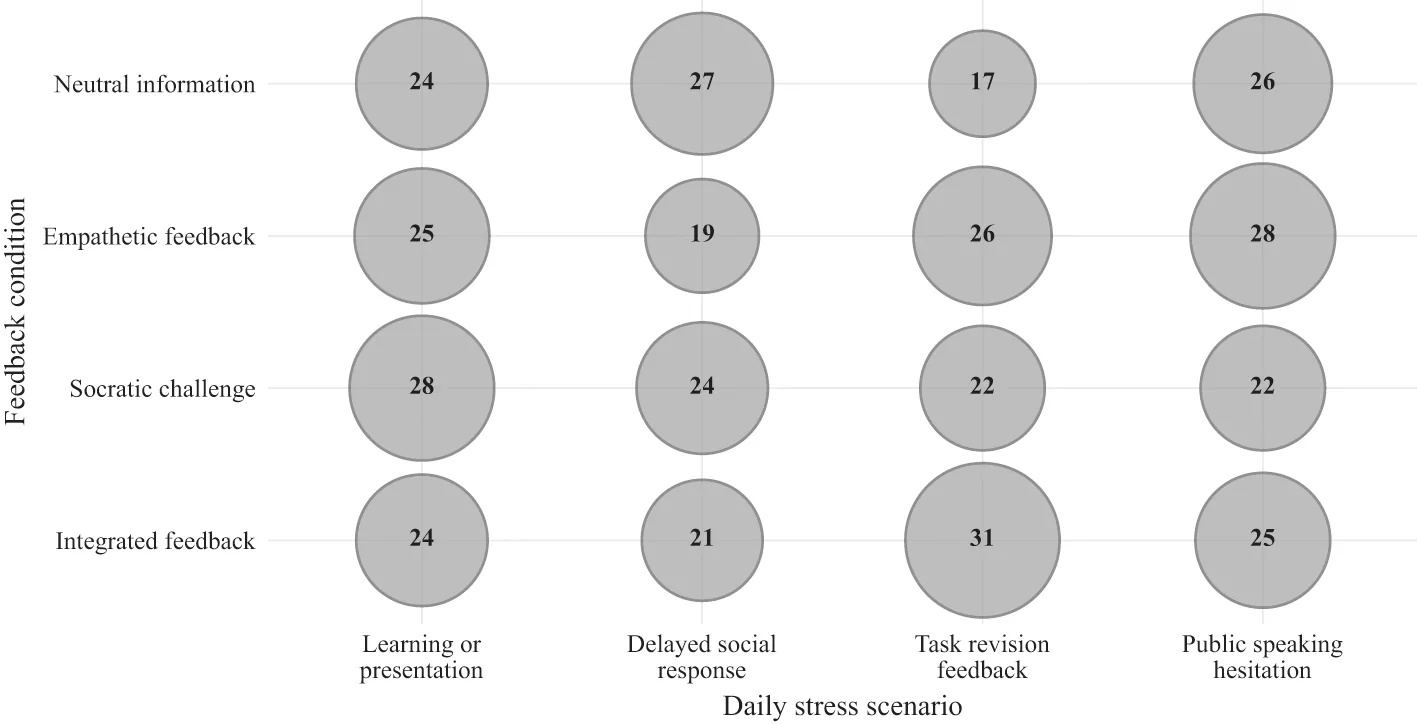
Valid participant counts by feedback condition and daily stress scenario. Bubble area is proportional to the cell count, and labels give the exact count.

### 4.3 Feedback generation and researcher review

Feedback texts were drafted through the official DeepSeek web interface before data collection, between February and March 2026. The research team used a logged-in account, did not enable DeepThink or web-search functions, and did not retain a verifiable record of the exact model build displayed by the interface. The platform is reported without assigning a retrospective model version. A separate conversation was initiated for each new scenario-generation task to reduce carryover across stimulus conditions. The same prompt framework was used across conditions and specified the scenario, target negative automatic thought, feedback condition, intended tone, audience, length, and prohibited content.

For each scenario × feedback-condition combination, approximately 3–5 candidate texts were generated. The research team selected and revised the final versions according to condition definition, language clarity, text length, tone intensity, and consistency with the intended feedback style. Revisions adjusted wording, controlled length and tone intensity, removed diagnostic or treatment-promissory language, and ensured that each text matched its prespecified operational definition. The resulting stimuli are best described as researcher-curated AI-drafted feedback fixed before data collection, which allowed feedback style to be compared under controlled stimulus conditions.

Text length was kept comparable across conditions. Feedback texts were controlled at approximately 95–130 English words in reporting length, corresponding to about 180–240 Chinese characters in the questionnaire materials. Average reporting lengths were 108 words for neutral information, 116 words for empathetic feedback, 121 words for Socratic challenge, and 128 words for integrated feedback. The platform record and stimulus-retention information are documented in Supplementary Table S1. The condition-specific prompt rules are documented in Supplementary Table S2, the fixed feedback stimuli are provided in Supplementary Table S4, and the retained prompt framework and participant-facing source statement are provided in Supplementary Methods S1–S2.

Two researchers with training in educational psychology independently reviewed all candidate texts. Stimulus control covered diagnostic wording, treatment promises, crisis-intervention instructions, excessive reassurance, direct denial of participants' feelings, Socratic challenge phrased as criticism or correction, evidence-testing components, and the absence of strong cognitive challenge in the empathetic condition. Disagreements were discussed until both reviewers agreed on the final stimuli. The condition definitions in Table 3 operationalize the intended stimulus contrast: neutral information served as the baseline comparison, empathetic feedback emphasized affective validation, Socratic challenge emphasized evidence testing, probability estimation, and fact-inference distinction, and integrated feedback used a written order in which validation preceded examination.

**Table 3:** Feedback conditions and design principles.

Feedback condition	Core function	Included components	Excluded components	Example sentence
Neutral information	General reflection	Description of stressful thoughts and reflection	Emotional validation; evidence testing; restructuring advice	“Stressful thoughts can be reviewed after the event.”
Empathetic feedback	Affective validation	Emotional acknowledgment; normalization; respectful tone	Strong evidence challenge; correction; probability testing	“It is understandable to feel upset after a setback.”
Socratic challenge	Evidence testing	Evidence testing; alternative explanation; probability estimation; fact-inference distinction	Blame; criticism; diagnostic claims; excessive reassurance	“What evidence supports this thought, and what evidence points to another explanation?”
Integrated feedback	Validation before examination	Emotional validation followed by evidence testing and balanced interpretation	Therapy promises; crisis instructions; direct invalidation; confrontational correction	“Your reaction makes sense; it may help to ask what evidence the thought uses.”

### 4.4 Measures

All scale items used 1–7 response options unless otherwise stated. Multi-item scores were calculated as item means according to Equation 1, with higher scores indicating a stronger level of the corresponding psychological characteristic or feedback evaluation.


$$\begin{array}{l} \bar{X}=\frac{1}{k}\overset{k}{\underset{i=1}{\sum }}x_{i} \end{array}$$ (1)

The self-developed items were reviewed before data collection by two researchers with psychology training. The review focused on construct relevance, wording clarity, item overlap, and potential clinical implication. Items with ambiguous wording, construct overlap, or clinical-risk phrasing were revised before the formal survey. Internal consistency was reported using Cronbach's alpha and McDonald's omega. These checks indicate content coverage and response consistency. Supplementary Table S5 provides the complete English item set, scoring instructions, and item-source notes.

NAT believability measured the degree to which participants believed the target negative automatic thought in the scenario. It included three pre-test items and the same three post-test items, and scores were computed as item means. The items were developed for this scenario-based task using the believability logic from automatic-thought measurement. An example item was “I would believe this thought reflects reality.” This construct served as the primary short-term cognitive outcome.

Negative affect measured immediate emotional response to the scenario and feedback. Four items assessed anxiety, sadness/depression, tension, and self-blame at pre-test and post-test. NAT believability and negative affect change scores were calculated as post-test minus pre-test values, as shown in Equations 2 and 3; negative values indicated a reduction.


$$\begin{array}{lll} \Delta \text{NAT} & = & NAT_{\text{post}}-\text{NA}{\text{T}}_{\text{pre}} \end{array}$$ (2)

$$\begin{array}{lll} \Delta \text{Affect} & = & Affect_{\text{post}}-\text{Affec}{\text{t}}_{\text{pre}} \end{array}$$ (3)

Perceived empathy and feedback acceptance were treated as feedback reception indicators. Perceived empathy included four self-developed items assessing whether the feedback made participants feel understood, accepted, and emotionally validated.

Feedback acceptance included four self-developed items measuring perceived usefulness, willingness to consider the feedback, and willingness to recommend similar feedback. Feedback reactance included four self-developed items measuring whether the feedback felt preachy, pressuring, disrespectful, or excessively challenging; higher scores indicated stronger reactance to the response. Cognitive reappraisal and reframing quality were treated as evidence-based thought-examination indicators. Cognitive reappraisal included four self-developed items assessing whether the feedback helped participants consider other explanations, distinguish facts from inferences, and form a balanced view. Trait NAT tendency included five items adapted from automatic-thought content to assess the tendency to generate negative automatic thoughts during daily setbacks. AI trust included three self-developed items assessing general trust in AI feedback or AI advice. Trait reactance included three self-developed items assessing resistance to external advice, persuasion, or challenge. Manipulation checks included two items assessing perceived empathy cues and Socratic-characteristic cues in the feedback. Table 4 groups variables as the primary cognitive outcome, affective outcome, feedback-reception indicators, evidence-based thought-examination indicators, and covariates. It also marks whether items were task-developed, self-developed, or adapted; the reported reliability coefficients (α = 0.73–.92, ω = 0.83–.94) support internal consistency and document the scoring procedure.

**Table 4:** Measures, scoring, and reliability.

Construct	Items	Source and scoring	Example item	Reliability
Trait NAT tendency	5	Adapted; item mean; higher = stronger tendency	“When facing setbacks, I quickly doubt my ability.”	α = 0.85, ω = 0.89
NAT believability	3+3	Task-developed; pre/post item means; higher = stronger belief	“I would believe this thought reflects reality.”	α = 0.81/.87, ω = 0.89/.92
Negative affect	4+4	Task-developed; change = post minus pre	“I would feel anxious.”	α = 0.84/.89, ω = 0.90/.93
Perceived empathy	4	Self-developed; item mean	“The feedback understood the person's feelings.”	α = 0.92, ω = 0.94
Cognitive reappraisal	4	Self-developed; item mean	“The feedback helped me see another interpretation.”	α = 0.92, ω = 0.94
Feedback acceptance	4	Self-developed; item mean	“I would consider using this feedback.”	α = 0.89, ω = 0.92
Feedback reactance	4	Self-developed; item mean	“The feedback felt preachy.”	α = 0.73, ω = 0.83
AI trust	3	Self-developed; item mean	“AI feedback can sometimes provide useful suggestions.”	α = 0.74, ω = 0.85
Trait reactance	3	Self-developed; item mean	“I dislike being told how to think about a problem.”	α = 0.80, ω = 0.88

### 4.5 Open-ended reframing coding

After reading the feedback, participants wrote a balanced alternative thought. Reframing quality was coded on a 0–3 scale to evaluate the quality of cognitive restructuring in the open-ended response. Two independent coders scored all responses and were blind to participants' feedback conditions. Before formal coding, the research team randomly selected 40 responses for training, discussed disagreements, and revised the coding manual. The full scoring rubric is reproduced in Supplementary Table S6.

A score of 0 indicated no alternative thought or simple repetition of the original negative thought. A score of 1 indicated simple reassurance or a vague positive statement, such as “Maybe it is not that bad.” A score of 2 indicated an alternative explanation with limited evidential grounding, such as “My friend may just be busy.” A score of 3 required evidence-based balanced reframing that distinguished facts from inferences, generated an alternative explanation, and reached a balanced conclusion. Interrater agreement was acceptable, with weighted kappa s= 0.78 and ICC(2,1) s= 0.82. Coding disagreements were resolved through discussion, and the consensus score was used in analysis.

### 4.6 Procedure and ethical safeguards

Participants first read an informed-consent statement. Before the scenario task, they saw a fixed source statement noting that the feedback text had been drafted with generative AI, reviewed and finalized by the research team, and presented according to the predefined experimental condition. Participants then read one randomly assigned daily stress scenario and completed pre-test measures of NAT believability and negative affect. Next, they read one fixed feedback text assigned independently from the scenario. They then completed post-test NAT believability, post-test negative affect, manipulation checks, perceived empathy, cognitive reappraisal, feedback acceptance, feedback reactance, and the open-ended alternative thought item. The final section collected demographic information and trait measures. The questionnaire did not include a separate source-perception manipulation check.

Participants provided informed consent before entering the questionnaire and could withdraw at any time without penalty. The survey was anonymous, used standardized hypothetical daily stress scenarios, and ended with an informational debriefing on the research purpose and the non-clinical use of the feedback materials. The questionnaire did not assess clinical diagnosis, treatment history, self-harm, or suicidal ideation. All records were de-identified before analysis. No separate list of psychological support services was provided at survey completion. The participant-facing source statement, consent information, and debriefing text are provided in Supplementary Methods S2–S3.

### 4.7 Data analysis

Analyses included descriptive statistics for sample characteristics, feedback conditions, and scenario distributions; internal-consistency analysis for multi-item scales; and manipulation checks for perceived empathy and Socratic cues. Baseline equivalence was examined using one-way ANOVA for pre-test NAT believability and pre-test negative affect.

The primary model was an ordinary least squares regression predicting post-test NAT believability. Feedback condition was entered as the independent variable with neutral information as the reference group. Covariates were pre-test NAT believability, scenario, trait NAT tendency, AI trust, and trait reactance. A parallel model predicted negative affect change while controlling for pre-test negative affect and the same covariates. Direct condition contrasts were tested from the adjusted models, and Holm-adjusted *p* values were used for the six pairwise condition contrasts within each primary outcome. Condition × scenario interactions were tested as exploratory models. Post-exposure process indicators were compared descriptively and with omnibus tests. Open-ended reframing quality was analyzed using descriptive proportions, a nonparametric omnibus comparison, and an ordinal logistic regression sensitivity analysis given the ordinal score.

## 5 Results

### 5.1 Descriptive statistics and baseline equivalence

The final analytic sample included 389 participants. Feedback condition sizes were 94 for neutral information, 98 for empathetic feedback, 96 for Socratic challenge, and 101 for integrated feedback. Scenario counts were 101 for learning or classroom presentation failure, 91 for delayed social response, 96 for task revision feedback, and 101 for public speaking hesitation. Returning to the condition-by-scenario distribution in Figure 2, all 16 randomized cells contributed observations to the analyses, while the unequal realized counts justify retaining scenario in the adjusted models.

Pre-test NAT believability did not differ significantly across feedback conditions, F_(3, 385)_ = 1.23, *p* = 0.298. Pre-test negative affect was also comparable, F_(_3, 385) = 0.41, *p* = 0.743. Thus, the later group differences were estimated from conditions that began at similar levels of target-thought credibility and negative affect; the regression models retained the relevant pre-test measure as a covariate to improve precision. The descriptive estimates in Table 5 give the exact means behind this baseline check and distinguish the two outcome patterns: NAT change followed the order integrated feedback, Socratic challenge, empathetic feedback, and neutral information, whereas affect change was larger for integrated and empathetic feedback than for Socratic challenge.

**Table 5:** Descriptive statistics by feedback condition.

Condition	*N*	NAT pre M(SD)	NAT post M(SD)	NAT change M(SD)	Affect change M(SD)
Neutral information	94	4.99 (0.74)	4.70 (0.79)	−0.29 (0.39)	−0.25 (0.40)
Empathetic feedback	98	4.83 (0.68)	4.25 (0.68)	−0.58 (0.42)	−0.74 (0.34)
Socratic challenge	96	4.83 (0.70)	4.07 (0.80)	−0.77 (0.43)	−0.51 (0.37)
Integrated feedback	101	4.83 (0.74)	3.83 (0.82)	−1.00 (0.47)	−0.83 (0.31)

The pre-post display in Figure 3 separates baseline comparability from post-feedback change: pre-test NAT believability was similar across conditions, whereas post-test scores declined most after integrated feedback, followed by Socratic challenge and empathetic feedback.

**Figure 3:**
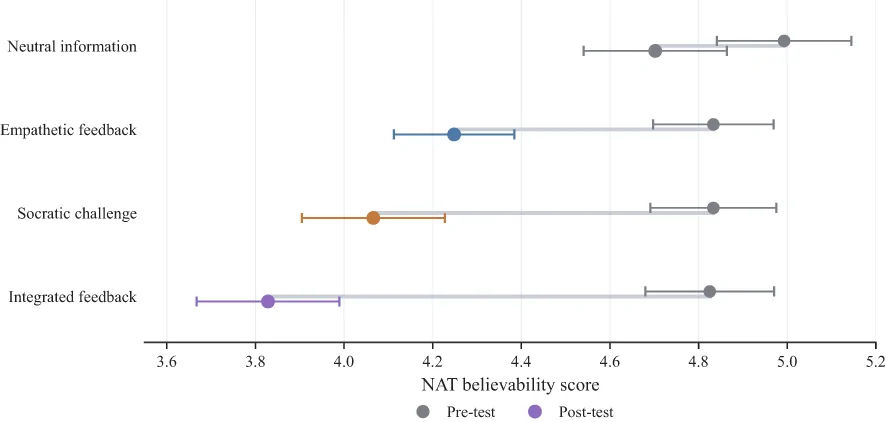
Pre- and post-test NAT believability by feedback condition. Points show condition means, horizontal error bars show 95% confidence intervals, and connecting lines indicate within-condition change.

### 5.2 Manipulation checks

Manipulation checks tested whether participants perceived the intended empathy and Socratic cues. Perceived empathy cue scores differed strongly across conditions, F_(_3, 385) = 283.80, *p* < 0.001. Empathetic feedback had the highest empathy cue score (*M* = 5.79, *SD* = 0.69), followed by integrated feedback (*M* = 5.49, *SD* = 0.67), Socratic challenge (*M* = 4.23, *SD* = 0.66), and neutral information (*M* = 3.37, *SD* = 0.59). This ordering is consistent with the intended emotional-validation manipulation.

Socratic cue scores also differed strongly, F_(_3, 385) = 492.09, *p* < 0.001. Socratic challenge had the highest score (*M* = 5.75, *SD* = 0.60), integrated feedback was also high (*M* = 5.30, *SD* = 0.52), and empathetic feedback (*M* = 3.38, *SD* = 0.68) and neutral information (*M* = 3.07, *SD* = 0.57) were lower. The two cue profiles in Figure 4 confirm the intended separation between validation-oriented and evidence-testing feedback cues, reducing the likelihood that later outcome differences reflect only general positivity or text readability.

**Figure 4:**
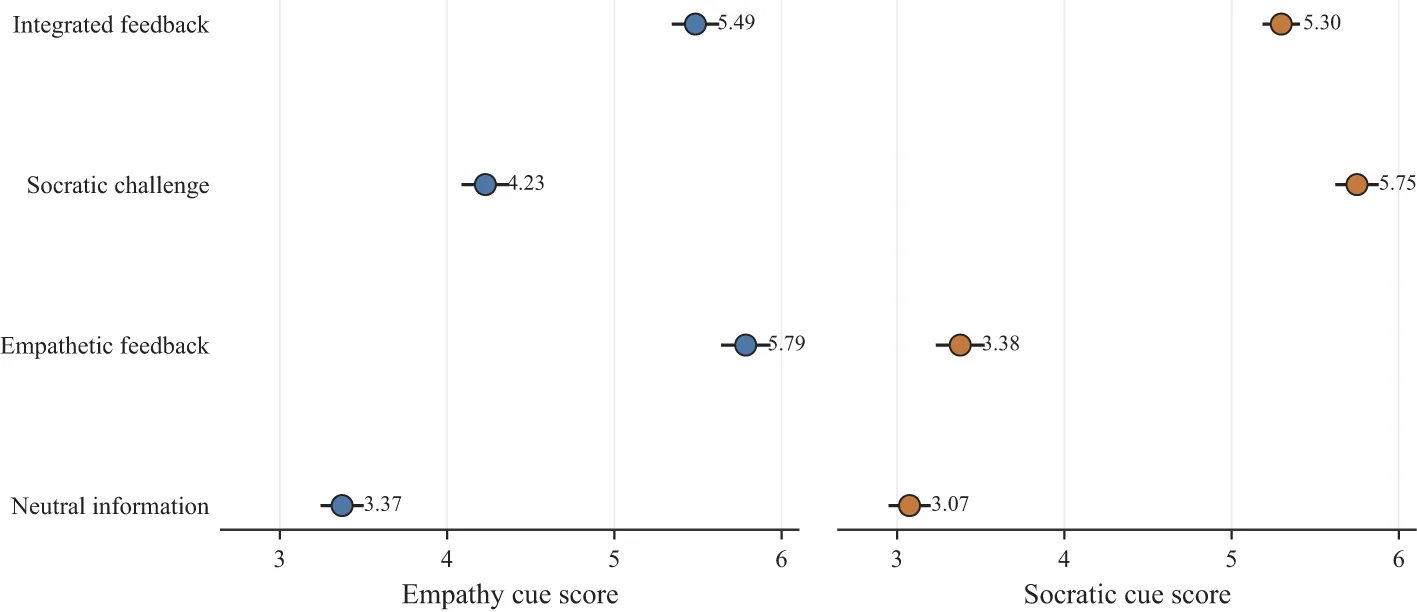
Manipulation check scores across feedback conditions. Points show condition means, horizontal error bars show 95% confidence intervals, and numeric labels report mean scores.

### 5.3 Effects on NAT believability and negative affect

The covariate-adjusted model in Table 6 explained a substantial proportion of variance in post-test NAT believability, *R*^2^ = 0.75. Relative to neutral information, empathetic feedback predicted lower post-test NAT believability, *B* = −0.31, *SE* = 0.06, 95% CI [−0.43, −0.19], *p* < 0.001. Socratic challenge showed a larger adjusted difference, *B* = −0.49, *SE* = 0.06, 95% CI [−0.62, −0.37], *p* < 0.001. Integrated feedback showed the largest adjusted difference, *B* = −0.73, *SE* = 0.06, 95% CI [−0.85, −0.61], *p* < 0.001.

**Table 6:** Full covariate-adjusted regression results for primary outcomes.

Outcome	Predictor	B	SE	95% CI	*p*
A. Post-test NAT believability					
NAT post believability	Empathy vs neutral	−0.31	0.06	[−0.43, −0.19]	< 0.001
NAT post believability	Socratic vs neutral	−0.49	0.06	[−0.62, −0.37]	< 0.001
NAT post believability	Integrated vs neutral	−0.73	0.06	[−0.85, −0.61]	< 0.001
NAT post believability	Pre-test believability	0.91	0.04	[0.83, 0.99]	< 0.001
NAT post believability	Scenario 2	0.07	0.06	[−0.06, 0.19]	= 0.288
NAT post believability	Scenario 3	0.10	0.06	[−0.02, 0.22]	= 0.112
NAT post believability	Scenario 4	0.08	0.06	[−0.03, 0.20]	= 0.164
NAT post believability	Trait NAT tendency	−0.01	0.04	[−0.09, 0.07]	= 0.756
NAT post believability	AI trust	−0.06	0.03	[−0.13, 0.00]	= 0.053
NAT post believability	Trait reactance	0.03	0.03	[−0.02, 0.09]	= 0.227
B. Negative affect change					
Negative affect change	Empathy vs neutral	−0.50	0.05	[−0.60, −0.40]	< 0.001
Negative affect change	Socratic vs neutral	−0.26	0.05	[−0.36, −0.16]	< 0.001
Negative affect change	Integrated vs neutral	−0.59	0.05	[−0.69, −0.49]	< 0.001
Negative affect change	Pre-test affect	−0.04	0.04	[−0.11, 0.03]	= 0.274
Negative affect change	Scenario 2	0.00	0.05	[−0.10, 0.10]	= 1.000
Negative affect change	Scenario 3	0.03	0.05	[−0.07, 0.13]	= 0.562
Negative affect change	Scenario 4	0.03	0.05	[−0.07, 0.13]	= 0.512
Negative affect change	Trait NAT tendency	0.01	0.04	[−0.05, 0.08]	= 0.674
Negative affect change	AI trust	−0.02	0.03	[−0.07, 0.04]	= 0.544
Negative affect change	Trait reactance	0.03	0.02	[−0.02, 0.07]	= 0.254

The negative affect change model explained 30% of the variance in affect change, *R*^2^ = 0.30. Empathetic feedback predicted greater affect reduction than neutral information, *B* = −0.50, *SE* = 0.05, 95% CI [−0.60, −0.40], *p* < 0.001. Socratic challenge also predicted greater affect reduction than neutral information, *B* = −0.26, *SE* = 0.05, 95% CI [−0.36, −0.16], *p* < 0.001. Integrated feedback showed the strongest affect reduction, *B* = −0.59, *SE* = 0.05, 95% CI [−0.69, −0.49], *p* < 0.001. Socratic challenge was stronger than empathetic feedback for NAT believability, whereas empathetic feedback was stronger than Socratic challenge for affect reduction, indicating different response patterns for belief credibility and emotional relief. The negative-affect coefficients align with H2 by placing empathetic feedback above Socratic challenge for affect reduction, while the integrated coefficients support H4 by combining the largest NAT-believability difference with the strongest affect reduction.

Holm-adjusted pairwise contrasts from the same models preserved the ordering among the active conditions: integrated feedback remained lower than empathetic feedback for NAT believability (*B* = −0.42, *SE* = 0.06, 95% CI [−0.54, −0.30], Holm-adjusted *p* < 0.001) and lower than Socratic challenge (*B* = −0.24, *SE* = 0.06, 95% CI [−0.36, −0.12], Holm-adjusted *p* < 0.001), while Socratic challenge remained lower than empathetic feedback (*B* = −0.18, *SE* = 0.06, 95% CI [−0.30, −0.06], Holm-adjusted *p* = 0.003). For negative affect change, integrated feedback remained lower than Socratic challenge (*B* = −0.33, *SE* = 0.05, 95% CI [−0.43, −0.23], Holm-adjusted *p* < 0.001), whereas the integrated-versus-empathetic contrast was smaller and did not survive Holm correction (*B* = −0.09, *SE* = 0.05, 95% CI [−0.19, 0.01], Holm-adjusted *p* = 0.074). The condition × scenario interaction was not supported for either outcome (NAT believability, F_(_9, 369) = 0.48, *p* = 0.887; affect change, F_(_9, 369)=0.88, *p* = 0.547), indicating that the adjusted ordering was consistent across the four scenarios.

### 5.4 Process indicators and reframing quality

The post-exposure process indicators followed different condition orderings. Perceived empathy was highest in the empathetic (*M* = 5.71, *SD* = 0.46) and integrated (*M* = 5.58, *SD* = 0.45) conditions, lower in the Socratic condition (*M* = 4.35, *SD* = 0.44), and lowest in the neutral condition (*M* = 3.56, *SD* = 0.44), F_(_3, 385) = 511.71, *p* < 0.001. Cognitive reappraisal was highest in the integrated condition (*M* = 5.75, *SD* = 0.42) and Socratic challenge condition (*M* = 5.55, *SD* = 0.46), followed by empathetic feedback (*M* = 4.79, *SD* = 0.46) and neutral information (*M* = 3.68, *SD* = 0.41), F_(_3, 385) = 437.80, *p* < 0.001. Empathetic wording was most closely tied to perceived understanding, whereas Socratic wording was more closely tied to evidence-based reconsideration of the target thought.

Feedback acceptance was highest for integrated feedback (*M* = 5.55, *SD* = 0.41), followed by empathetic feedback (*M* = 5.38, *SD* = 0.40), Socratic challenge (*M* = 4.80, *SD* = 0.40), and neutral information (*M* = 3.64, *SD* = 0.45), F_(_3, 385) = 413.17, *p* < 0.001. Feedback reactance was highest in the Socratic challenge condition (*M* = 3.41, *SD* = 0.46), followed by neutral information (*M* = 3.00, *SD* = 0.58), integrated feedback (*M* = 2.84, *SD* = 0.45), and empathetic feedback (*M* = 2.67, *SD* = 0.44), F_(_3, 385) = 40.74, *p* < 0.001. The higher cognitive reappraisal score for Socratic challenge than for empathetic feedback is consistent with H3, although the same condition also produced higher perceived pressure and reactance; integrated feedback retained high reappraisal while keeping reactance closer to the empathetic condition.

Open-ended reframing quality also differed across groups. Mean reframing quality was lowest in the neutral condition (*M* = 1.40, *SD* = 0.71), higher in the empathetic condition (*M* = 1.80, *SD* = 0.72), higher again in the Socratic condition (*M* = 2.02, *SD* = 0.79), and highest in the integrated condition (*M* = 2.34, *SD* = 0.72). A nonparametric comparison supported an omnibus group difference, Kruskal-Wallis *H* = 67.83, *p* < 0.001. The proportional distribution in Figure 5 adds open-text evidence for H3 by showing how often participants produced higher-quality alternative thoughts. Score-3 responses required fact-inference distinction, an alternative explanation, and a balanced conclusion; their larger share in the integrated condition indicates that validation followed by evidence testing was more likely to translate into evidence-based reframing. Socratic challenge also produced more score-3 responses than empathetic feedback, but this cognitive gain occurred alongside higher feedback reactance. An ordinal logistic regression sensitivity analysis with condition, scenario, pre-test NAT believability, trait NAT tendency, AI trust, and trait reactance produced the same ordering: relative to neutral information, the odds of higher reframing quality were greater for empathetic feedback (OR = 2.76, *p* < 0.001), Socratic challenge (OR = 4.85, *p* < 0.001), and integrated feedback (OR = 11.12, *p* < 0.001). Direct ordinal contrasts also favored integrated feedback over Socratic challenge (OR = 2.29, *p* = 0.003) and empathetic feedback (OR = 4.03, *p* < 0.001), and Socratic challenge exceeded empathetic feedback (OR = 1.75, *p* = 0.038).

**Figure 5:**
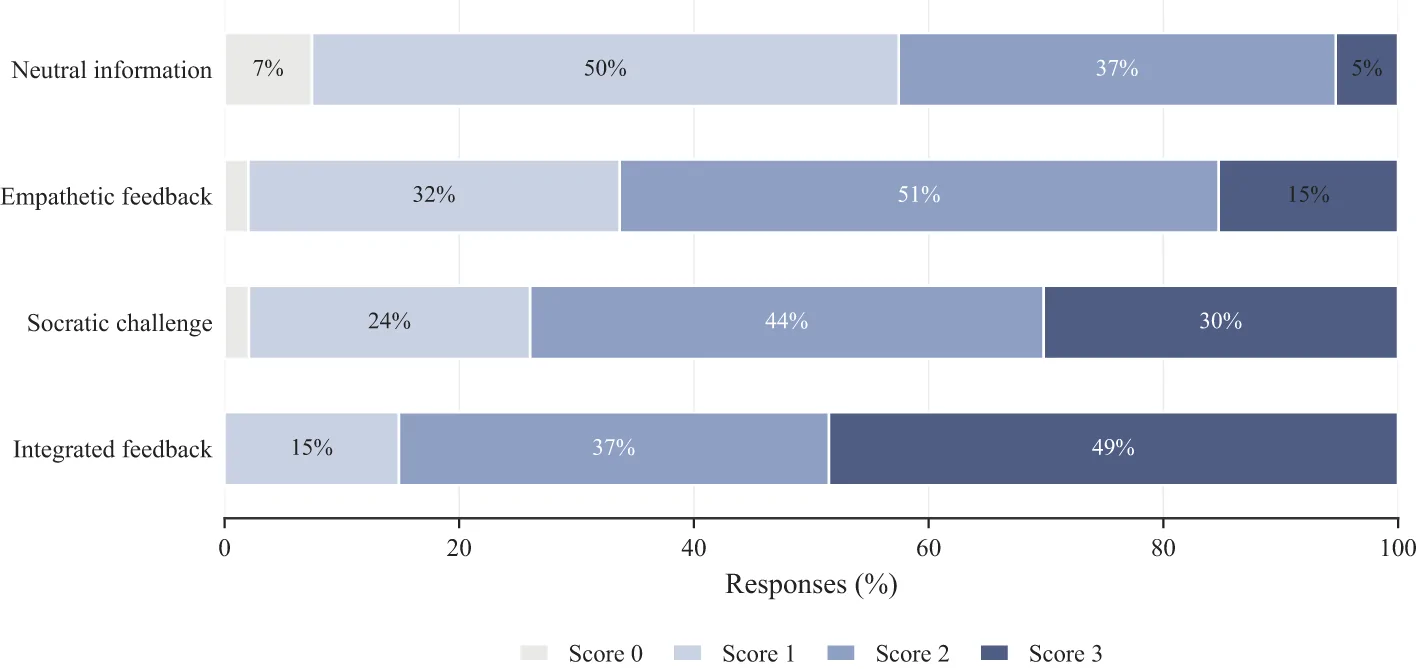
Stacked percentage distribution of reframing quality scores by feedback condition. Score 3 represents evidence-based balanced reframing that includes fact-inference distinction, an alternative explanation, and a balanced conclusion. Percentages are rounded within condition; score-0 segments are retained where present.

## 6 Discussion

The evidence from manipulation checks, descriptive statistics, adjusted regressions, and open-ended coding converges on one pattern of feedback style. Participants perceived the intended validation and evidence-testing cues; the active feedback conditions reduced NAT believability more than neutral information; and the integrated condition paired the largest reduction in belief credibility with strong affect reduction, feedback acceptance, cognitive reappraisal, and evidence-based balanced reframing. The open-ended coding adds another layer by linking rating results to participants' own alternative thoughts.

Empathetic feedback primarily changed how participants received the message. It produced high perceived empathy, high feedback acceptance, low feedback reactance, and stronger affect reduction than Socratic challenge. This result accords with supportive communication research showing that validation and being understood make difficult feedback easier to consider (Rogers, 1957; Elliott et al., 2011; Norcross and Lambert, 2019). Its lower reframing quality relative to Socratic challenge and integrated feedback indicates that emotional validation alone was less likely to elicit fact-inference distinction or a well-supported alternative explanation.

Socratic challenge was more closely tied to change in the credibility of the target thought. It reduced NAT believability more than empathetic feedback and produced higher cognitive reappraisal and reframing quality, indicating that evidence testing, probability estimation, and alternative explanation helped participants inspect the negative interpretation (Clark and Egan, 2015; Braun et al., 2015; Held et al., 2024, 2025). The same condition also produced the highest feedback reactance, suggesting that a direct request to examine evidence can feel pressuring even when it supports belief revision. For researcher-curated AI-drafted cognitive restructuring feedback, Socratic wording needs to preserve inquiry and avoid sounding like correction.

Integrated feedback combined validation and evidence testing in one text. The data are consistent with that written order, but the design did not isolate sequence from content combination. The result profile links that combined format to strong affect reduction, high feedback acceptance, high cognitive reappraisal, and the highest reframing quality. Its larger proportion of score-3 responses matters, since the coding category required evidence-based balanced reframing with more than simple reassurance.

Participants were recruited through Wenjuanxing and university links, and most were young adults with undergraduate or graduate education. They read one fixed feedback text after a hypothetical daily-stress scenario, so repeated conversational use and self-generated thoughts may produce different response patterns. The open-ended reframing task came immediately after feedback, and neutral information served as the comparison baseline. The design did not include a blank no-feedback condition. The self-developed scales were content-reviewed and internally consistent; external validation and a separate source-perception check remain useful next steps. Future studies can extend the design with diary materials, follow-up measurement, reverse-order integrated texts, human counselor comparisons, different AI models, and cross-cultural samples.

## 7 Conclusion

This online randomized scenario-based experiment compared four researcher-curated AI-drafted feedback styles and found that feedback style shaped NAT believability, negative affect, feedback acceptance, feedback reactance, cognitive reappraisal, and reframing quality after a single exposure. Empathetic feedback supported perceived empathy, acceptance, lower reactance, and affect reduction; Socratic challenge more strongly supported cognitive reappraisal and evidence-based reframing. Integrated feedback combined these advantages by first acknowledging the emotional response and then guiding evidence testing, alternative explanation, and fact-inference distinction. For daily reflection tasks involving negative automatic thoughts, feedback design is therefore more than a matter of warm versus challenging tone: emotional validation can make the response easier to receive, and evidence testing can help participants produce a more balanced interpretation.

## Data Availability

The raw data supporting the conclusions of this article will be made available by the authors, without undue reservation.
