# Supplementary material for: Comparing researcher-curated AI-drafted feedback styles for negative automatic thought believability: an online randomized scenario-based experiment
Source: Front Psychol. 2026 Oct 1;17:1926092. doi: 10.3389/fpsyg.2026.1926092 (PMC13669167; doi:10.3389/fpsyg.2026.1926092)
Supplement: Supplementary file 1 [file Data_Sheet_1.pdf]

Supplementary Material

1 SUPPLEMENTARY METHODS

1.1 S1. AI system and stimulus-generation record

Feedback texts were drafted through the official DeepSeek web interface (`https://chat.deepseek.com/`) between February and March 2026, before formal data collection. A logged-in account was used. DeepThink and web-search functions were not enabled. The research team did not retain a verifiable record of the exact model build displayed by the web interface and therefore reports the platform rather than assigning a retrospective model version. A new conversation was initiated for each scenario-generation task. Approximately 3–5 candidate texts were generated for each scenario-by-feedback-condition combination. The final texts were selected and revised by the research team. All final adopted texts were retained.

Supplementary Table S1 lists the generation settings and retention status for the stimulus procedure.

Table S1. AI system and stimulus-generation record.

Item	Recorded information
Platform	Official DeepSeek web interface
Generation period	February–March 2026, before formal data collection
Model version	Exact model build not verifiably retained
Account status	Logged-in account
DeepThink	Not enabled
Web search	Not enabled
Conversation management	A new conversation was initiated for each scenario-generation task
Candidate generation	Approximately 3–5 candidates per scenario-by-condition combination
Human review	Selection and revision by two researchers with educational-psychology training
Main revisions	Language clarity, length, tone intensity, condition alignment, and removal of diagnostic or treatment-promissory wording
Retention	All final adopted texts retained

1.2 S2. Retained prompt framework and source statement

The complete verbatim prompt for every generation was not retained. The research team retained the common prompt framework and the final adopted stimulus texts. The framework specified the scenario, target negative automatic thought, feedback condition, intended tone, audience, length, and prohibited content.

```
Please generate a Chinese feedback text for a psychology questionnaire
based on the daily stress scenario and target negative automatic thought.

Scenario: [insert scenario]
Target negative automatic thought: [insert target thought]
Feedback condition: [neutral / empathetic / Socratic / integrated]

The text is for an online scenario-based experiment with adults. It is for
research use and is not intended for psychological diagnosis or treatment.
Use natural, clear, and respectful language. Do not use diagnostic language,
treatment promises, crisis-intervention instructions, or exaggerated
guarantees. Keep the text at approximately 180-240 Chinese characters.

Neutral information: provide general information about the stressful event
and reflection; do not add strong reassurance, evidence testing, or explicit
cognitive restructuring.

Empathetic feedback: acknowledge and normalize the emotional response,
express respect, and avoid strong evidence challenge, probability estimation,
or direct correction.

Socratic challenge: ask the participant to examine evidence for and against
the thought, distinguish facts from inferences, consider alternative
explanations, and evaluate plausibility. Avoid criticism, blame, and
emotional invalidation.

Integrated feedback: acknowledge and understand the emotional response first,
then guide evidence testing, fact-inference distinction, alternative
explanation, and a more balanced judgment.

Output only the feedback text.
```

Supplementary Table S2 records the condition-specific prompt rules used to keep the four feedback styles distinguishable.

Table S2. Condition-specific prompt rules.

Condition	Core function	Included components	Excluded components
Neutral information	General reflection	General description of the stressful event and reflection process	Strong emotional reassurance; direct evidence testing; explicit restructuring advice
Empathetic feedback	Affective validation	Emotional acknowledgment; normalization; respectful tone	Strong evidence challenge; probability testing; direct correction
Socratic challenge	Evidence testing	Evidence for and against the thought; alternative explanation; probability estimation; fact-inference distinction	Blame; criticism; emotional invalidation; diagnostic claims
Integrated feedback	Validation before evidence testing	Emotional acknowledgment followed by evidence testing and balanced interpretation	Treatment promises; crisis instructions; confrontational correction

The same participant-facing source statement appeared across conditions:

> The following response is a feedback text prepared for research use. The text was drafted with the assistance of a generative AI system and was reviewed and finalized by the research team before being used in the study. Feedback materials were presented according to predefined experimental conditions. Please answer the following questions based on your actual response to the text.

1.3 S3. Consent and debriefing materials

The questionnaire informed participants that the study concerned evaluations of daily stress scenarios and feedback texts, that participation was voluntary, that participants could withdraw before submitting the questionnaire, and that the survey did not request direct identifying information. The scenarios were hypothetical and did not assess clinical diagnosis, treatment history, self-harm, or suicidal ideation.

The survey ended with an informational debriefing describing the four feedback styles and their research purpose. Participants were told that the materials were not psychological diagnosis, treatment, or individualized advice. No separate list of psychological support services was provided at survey completion.

2 SUPPLEMENTARY TABLES

2.1 S4. Scenario materials

Supplementary Table S3 links each scenario to its trigger, target thought, and psychological focus. The wording below reconstructs the scenario text used in the experiment.

Table S3. Daily stress scenarios and target negative automatic thoughts.

Scenario	Situational trigger	Target negative automatic thought	Psychological focus
Learning or classroom presentation failure	Unsuccessful learning task or classroom presentation	"I am not capable enough, and this failure shows I will have difficulty doing well in the future."	Academic ability doubt after setback
Delayed social response	Message remains unanswered longer than expected	"The other person may no longer care about me or may be deliberately distancing themselves from me."	Ambiguous social-rejection inference
Task revision feedback	Teacher or peer requests substantial revision	"So many revision comments show that my work is poor and that I am not capable enough."	Competence-related self-evaluation
Public speaking hesitation	Noticeable pause during public speaking	"Everyone must think that I performed very poorly."	Public-evaluation concern

2.2 S5. Feedback stimuli

Supplementary Table S4 provides the 16 fixed feedback stimuli by scenario and condition. The table includes the Chinese stimulus text and an English reference translation for each scenario-by-condition cell.

Table S4: Feedback stimuli by scenario and condition.

Scenario and condition	Chinese stimulus text	English reference translation
S1 Neutral information	一次学习任务或课堂展示没有达到预期，并不能单独说明一个人的整体能力。人在经历不理想的结果后，常会重新回顾准备过程、现场表现和结果，并据此形成对自身能力的判断。这类判断可以在情绪稍微稳定后重新查看，把这一次具体表现、任务难度、准备情况和对长期能力的总体评价分别考虑。一次结果通常只能提供关于当次表现的信息，是否能推及更稳定的能力，需要结合更多经历和信息来判断。	An important learning task or classroom presentation that falls short of expectations does not by itself describe a person's overall ability. After an unsatisfactory result, people often review their preparation, performance, and outcome and use these details to judge their ability. The event can be revisited after the initial reaction has settled by considering the specific performance, task difficulty, preparation, and broader evaluation separately. One result provides information about that occasion; whether it reflects stable ability requires more experiences and evidence.

Scenario and condition	Chinese stimulus text	English reference translation
S1 Empathetic feedback	认真准备之后却没有得到理想结果，感到失望、沮丧，甚至开始怀疑自己，是很容易出现的反应。尤其当你很重视学习表现时，一次没有发挥好会显得格外突出。现在的不舒服并不奇怪，也没有必要马上要求自己变得积极。先承认这次经历确实让你受挫，并给自己一点时间消化这种感受，是可以理解的。一次令人失望的经历也可能让人暂时只看到不足，而忽略之前的努力，这种情绪反应本身值得被认真对待。	Preparing carefully and then receiving a result that falls short of expectations can naturally bring disappointment, frustration, or self-doubt. When learning performance matters to you, one difficult experience may stand out and feel especially important. The discomfort does not need to be pushed away immediately, and there is no need to force yourself to feel positive. Acknowledging that the experience was discouraging and giving yourself time to absorb that feeling is understandable. A disappointing experience can temporarily highlight shortcomings and obscure earlier effort, and that emotional reaction deserves attention.
S1 Socratic challenge	可以检查一下"这次失败说明我能力不够"这个判断目前有哪些证据支持。一次任务表现不理想，是否足以代表整体能力？过去是否有完成得较好的任务？这一次还可能受到准备时间、题目难度、紧张程度或表达状态的影响吗？哪些是已经发生的事实，哪些是从一次结果进一步推断出的结论？如果同样的事情发生在别人身上，你会作出相同判断吗？还可以比较支持这个想法和不支持这个想法的证据，再判断"能力不够"是否是目前唯一合理的解释。	Consider what evidence supports the judgment that this failure shows insufficient ability. Is one unsatisfactory task enough to represent overall ability? Have there been tasks that went well, and could preparation time, task difficulty, nervousness, or expression have affected this performance? Which parts are facts that occurred, and which parts are conclusions inferred from one result? If the same event happened to someone else, would you reach the same judgment? Comparing evidence for and against the thought may show whether lack of ability is the only plausible explanation.
S1 Integrated feedback	认真准备后却没有达到自己的预期，感到失望和怀疑自己是可以理解的。这样的结果确实容易让人把注意力集中到不足上，也可能让之前的努力显得不那么重要。与此同时，可以再看看"这次失败说明我能力不够"这个判断有多少证据支持。一次表现是否能够代表长期能力？是否还存在准备、任务难度或紧张等其他解释？把已经发生的事实和由此作出的推断分开，再同时看看支持与不支持这个判断的证据，可能会形成更平衡的看法。	Preparing carefully and falling short of your expectations can understandably bring disappointment and self-doubt. A result like this can draw attention to shortcomings while making earlier effort less visible. At the same time, it may help to examine how much evidence supports the thought that this failure shows insufficient ability. Can one performance represent long-term ability, or could preparation, task difficulty, or nervousness also have contributed? Separating what happened from the conclusion drawn from it, and considering evidence on both sides, may support a more balanced judgment.

Scenario and condition	Chinese stimulus text	English reference translation
S2 Neutral information	日常交流中的回复速度会受到时间安排、学习或工作状态、设备使用习惯等多种因素影响。当消息没有及时得到回应时，人们往往会根据有限的信息推测对方的态度。重新看待这类情况时，可以把目前已经确认的事实、尚未获得的信息以及自己对对方意图的解释分别列出来，再判断现有信息能够支持到什么程度。延迟回复本身是一项可观察的信息，而对关系状态和对方动机的判断通常还需要更多直接线索。	Reply speed in everyday communication can reflect schedules, work or study demands, device use, and personal habits. When a message is not answered promptly, people often infer the other person’s attitude from limited information. Reviewing the situation can involve separating confirmed facts, information that is still unavailable, and interpretations of the other person’s intention. A delayed reply is observable information, whereas a conclusion about the state of a relationship or the other person’s motive generally requires additional direct evidence.
S2 Empathetic feedback	等待一个自己在意的人回复，而迟迟没有收到消息，确实很容易让人不安，也可能让人担心关系是不是发生了变化。尤其过去对方通常回复较快时，这一次的沉默会显得更加明显。出现失落、担心或反复猜测的感觉是可以理解的，也不需要因为自己产生这些想法而责怪自己。先承认这种等待带来的不确定感，是很自然的反应。关系对你越重要，暂时没有回应时产生的情绪波动越容易被放大，这种感受本身并不需要被否定。	Waiting for a reply from someone who matters to you can feel unsettling, especially when the person usually responds quickly. The silence may make you worry that the relationship has changed, and feelings of disappointment, concern, or repeated guessing are understandable in that situation. You do not need to blame yourself for having these reactions. Acknowledging the uncertainty created by the wait is a natural first step. The more important the relationship is, the more noticeable a temporary lack of response may feel, and the feeling itself does not need to be denied.
S2 Socratic challenge	可以检查一下“对方不在乎我或正在疏远我”这个判断目前有哪些直接证据。没有及时回复是否只有这一种解释？对方是否可能正在忙、忘记回复、没有精力交流，或者暂时不方便回应？“消息还没有回复”是已经知道的事实，而“对方正在拒绝我”属于事实还是推断？在获得更多信息之前，哪一种解释与现有证据更匹配？还可以想一想，过去对方有没有出现过延迟回复但关系并未发生变化的情况，这些信息是否会改变目前的判断。	Examine the direct evidence for the judgment that the other person does not care or is distancing themselves. Is a delayed reply the only possible explanation? Could the person be busy, have forgotten, lack the energy to talk, or be temporarily unable to respond? The fact that the message has not been answered is known, but is the conclusion that the person is rejecting you a fact or an inference? Before more information is available, which explanation fits the evidence better? Past occasions of delayed replies without a change in the relationship may also be relevant.

Scenario and condition	Chinese stimulus text	English reference translation
S2 Integrated feedback	在意的人没有像平时一样及时回复，感到不安或担心关系发生变化是可以理解的。等待本身很容易让人反复猜测对方的态度，尤其当这段关系对你比较重要时。不过，目前能够确认的事实只是消息暂时没有得到回复。对方是否不在乎你，还存在许多其他可能解释，例如忙碌、分心或暂时不方便交流。把“没有回复”和“正在拒绝我”区分开，再看看过去的互动以及后续是否出现新的信息，可能比立即确定对方的动机更接近现有证据。	When someone important does not reply as quickly as usual, it is understandable to feel uneasy or worry that the relationship has changed. Waiting can invite repeated guesses about the other person's attitude, particularly when the relationship matters to you. At present, however, the confirmed fact is that the message has not received a reply. There are several other possible explanations, including busyness, distraction, or temporary unavailability. Separating no reply from deliberate rejection, and considering past interactions and later information, may fit the evidence better than deciding the person's motive immediately.
S3 Neutral information	收到较多修改意见时，人们常会把注意力放在需要改动的部分，并据此重新评价自己的作品。修改意见本身通常说明当前文本、方案或任务中存在可以完善的地方，但它与对个人整体能力的评价并不是同一层面。重新查看反馈时，可以区分具体需要修改的内容、反馈者提出的要求，以及自己由这些意见延伸出的总体判断。反馈的数量也不一定直接等于作品质量，它可能受到任务要求、修改阶段和反馈者标准等因素影响。	When a completed task receives many revision comments, attention often turns to the parts that need to change, and people may use those comments to reassess their work. Revision requests usually identify features that can be improved; they are not equivalent to an evaluation of a person's overall ability. The feedback can be reviewed by separating the specific changes requested, the standards applied by the reviewer, and any broader judgment inferred from the comments. The number of revisions may also reflect task requirements, the stage of the work, or the reviewer's standards rather than work quality alone.
S3 Empathetic feedback	完成一项自己投入了时间的任务后，却看到很多修改意见，感到挫败、失望或怀疑自己的能力是很容易出现的。尤其当你原本认为完成得还不错时，密集的反馈会让这种落差更明显。你不需要因为这些感受而责怪自己，也不用马上否定自己的努力。先承认收到大量修改意见确实会让人不好受，是一种可以理解的反应。认真投入之后却发现还有很多地方需要调整，可能会让人暂时觉得之前的努力没有得到认可，这种失落感值得被看到。	After investing time in a task, seeing many revision comments can understandably feel discouraging, disappointing, or threatening to your confidence. The contrast may be especially difficult when you had believed that the work was generally successful. You do not need to blame yourself for these feelings or immediately dismiss the effort you made. Acknowledging that substantial feedback can be difficult to receive gives the disappointment room to be recognized. When a task requires more adjustment than expected, it is natural to feel that the earlier effort was overlooked, and that sense of loss deserves attention.

Scenario and condition	Chinese stimulus text	English reference translation
S3 Socratic challenge	可以检查一下“修改意见很多，所以我的作品很差、我也不够有能力”这个判断是否超出了现有证据。反馈中哪些内容是在指出具体问题，哪些地方其实被保留或认可？需要修改是否等同于整体失败？老师或同伴提出修改，也可能意味着任务还有提升空间。把“作品需要修改”和“我整体没有能力”分开，这两个结论分别有哪些证据支持？如果同样数量的修改意见出现在别人的作品中，你是否也会据此判断那个人整体没有能力？	Examine whether the judgment that many revision comments prove poor work and insufficient ability goes beyond the available evidence. Which comments identify specific problems, and which parts of the work were retained or recognized? Does a need for revision amount to overall failure? A teacher or peer may request changes because the task has room for improvement or because a particular standard is being applied. How much evidence supports the separate conclusions that the work needs revision and that you lack ability? If the same number of comments appeared on another person’s work, would you draw the same conclusion about that person’s overall ability?
S3 Integrated feedback	看到一项自己认真完成的任务被提出很多修改意见，感到失望或怀疑自己是可以理解的，尤其当你原本期待得到更积极的评价时。大量修改建议很容易让人首先注意自己的不足。与此同时，可以进一步看看这些意见实际说明了什么：它们是在指出具体需要改进的内容，还是能够证明你的整体能力不足？需要修改并不自动等于整体失败。把具体反馈与对自身能力的总体评价分开，再看看哪些部分被认可、哪些部分需要调整，可能有助于形成更符合现有证据的判断。	Seeing many revision comments on a task you completed carefully can understandably bring disappointment or self-doubt, especially when you expected a more positive evaluation. A large amount of feedback can make shortcomings stand out first. It may help to ask what the comments actually show: do they identify specific parts that need improvement, or do they establish that your overall ability is inadequate? A need for revision does not automatically mean that the whole task failed. Separating the concrete feedback from the broader judgment about your ability, while considering both recognized and changeable parts, may produce a conclusion that better fits the evidence.

Scenario and condition	Chinese stimulus text	English reference translation
S4 Neutral information	公开表达过程中出现停顿或短暂失去流畅性，是一种可以发生在多种情境中的表现。听众可能注意到停顿，也可能更关注发言的整体内容。人在结束发言后，往往会反复回想自己最不满意的部分，并据此推测他人的评价。重新回顾时，可以把实际发生的表现、自己注意到的细节和对他人想法的推测分别看待。一次停顿能够说明当时出现了表达中断，但并不能直接提供听众对整段发言如何评价的信息。	A pause or brief loss of fluency during public speaking can occur in many situations. Listeners may notice the pause, or they may attend more to the content of the talk as a whole. After speaking, people often replay the part they disliked most and use it to infer how others evaluated them. The event can be reviewed by separating the observable performance, the details that drew your attention, and guesses about the audience’s thoughts. A pause provides information about an interruption in expression, but it does not directly reveal how the audience judged the entire talk.
S4 Empathetic feedback	在公开场合说话时突然停顿，之后一直想到刚才那一刻，感到尴尬、紧张或担心别人怎么看自己，是很容易理解的。公开表达本身就会让人更加关注自己的表现，一个明显的停顿也可能被自己放大。即使你之后完成了发言，那一刻仍然可能让人觉得不舒服。先承认这种尴尬和担心是真实的感受，不需要马上强迫自己忽略它。人在被很多人注视时通常会更敏感地注意自己的失误，因此结束后反复想到那个片段也是一种常见的情绪反应。	Pausing noticeably while speaking in public and then continuing to think about that moment can understandably bring embarrassment, tension, or concern about how others saw you. Public speaking already directs attention toward one’s performance, so a pause may feel larger from the speaker’s perspective. Even after completing the talk, the moment can remain uncomfortable. You do not need to force yourself to ignore that reaction. When many people are watching, it is common to notice one’s own mistake closely and replay it afterward; that emotional response deserves recognition.
S4 Socratic challenge	可以检查一下“别人一定觉得我表现得很差”这个判断目前有哪些证据。你知道听众实际怎么评价吗，还是主要根据自己的停顿作出推测？一次停顿是否足以代表整段发言？听众也可能把它理解为紧张、思考或普通的表达卡顿。哪些是你确实观察到的事实，哪些是关于别人想法的推断？如果没有直接反馈，现有证据能支持“别人一定这样认为”吗？还可以回想发言的其他部分是否顺利，以及是否有人表现出明确的负面反应，再判断目前的结论有多确定。	Examine the evidence for the judgment that everyone thought the performance was very poor. Do you know how the audience evaluated the talk, or are you inferring their view mainly from the pause? Is one pause enough to represent the entire presentation? Listeners might interpret it as nervousness, thinking, or an ordinary moment of hesitation. Which parts are directly observed, and which parts are inferences about other people’s thoughts? Without direct feedback, can the available evidence support the certainty of the word “everyone”? The rest of the talk and any clear audience reactions may also matter.

Scenario and condition	Chinese stimulus text	English reference translation
S4 Integrated feedback	公开发言时突然停顿，感到尴尬并担心别人如何评价自己是可以理解的，尤其在很多人注视的情况下，这一刻很容易被自己反复回想。一个明显的停顿确实可能让人对自己的表现格外敏感。同时，可以再看看“别人一定觉得我表现得很差”这个判断依据是什么。你是否真的知道听众的评价？一次停顿是否代表整段发言？别人也可能把它看作紧张、思考或普通卡顿。把实际发生的停顿和对他人想法的推测分开，再结合整段表现判断，可能更接近现有证据。	Pausing during a public talk can understandably feel embarrassing and can make you worry about how others evaluated you, especially when many people were watching. The moment may remain vivid after the talk and draw attention away from parts that went smoothly. It may help to examine the basis for the thought that everyone thought you performed very poorly. Do you know the audience's evaluation, or are you inferring it from one pause? Others may have understood the pause as nervousness, thinking, or an ordinary hesitation. Separating the pause from the interpretation of other people's thoughts, and considering the whole talk, may fit the available evidence better.

2.3 S6. Questionnaire items and scoring

Supplementary Table S5 provides the complete English item set used for scoring. All items used a 1–7 response scale unless otherwise noted. Scores were calculated as item means.

Table S5: Questionnaire items and scoring rules.

Construct	Items	Scoring
NAT believability	I would believe that this thought reflects reality; I would think that this thought is probably true; In this situation, I would find this thought convincing.	Three-item mean at pre-test and post-test; higher = stronger belief.
Negative affect	I would feel anxious; I would feel sad or emotionally low; I would feel tense; I would blame myself.	Four-item mean at pre-test and post-test; change = post minus pre.
Perceived empathy	The feedback understood the person's feelings; the feedback acknowledged the person's emotional response; the feedback made the person's feelings seem respected; the feedback was emotionally supportive.	Four-item mean; higher = stronger perceived empathy.
Cognitive reappraisal	The feedback helped me see other possible explanations; distinguish facts from my inferences; reevaluate the original negative thought; form a more balanced view.	Four-item mean; higher = stronger reappraisal.

Construct	Items	Scoring
Feedback acceptance	I considered this feedback useful; I was willing to consider what this feedback said; I would refer to this type of feedback in a similar situation; I would recommend this type of feedback to someone in a similar situation.	Four-item mean; higher = stronger acceptance.
Feedback reactance	The feedback felt preachy; the feedback made me feel pressured to accept a particular view; the feedback made me feel that my feelings were not fully respected; the feedback placed too strong a demand on changing my thought.	Four-item mean; higher = stronger reactance.
Trait NAT tendency	When facing setbacks, I quickly begin to doubt my ability; when things do not go as planned, I often think about the worst possible outcome; negative evaluation is easily interpreted as rejection of my overall ability; ambiguous social situations invite negative interpretations; one poor performance is taken as evidence of future similar outcomes.	Five-item mean; higher = stronger tendency.
AI trust	AI feedback can sometimes provide useful suggestions; in some daily-life problems, I am willing to consider feedback from AI; AI-generated suggestions can be useful in some situations.	Three-item mean; higher = stronger trust.
Trait reactance	I dislike being told how I should view a problem; I usually resist when someone tries to change my thinking; when facing obvious persuasion or challenge, I tend to hold more strongly to my original view.	Three-item mean; higher = stronger trait reactance.
Manipulation checks	This feedback showed understanding and acceptance of the person's emotions; this feedback encouraged the person to examine evidence and rethink the original judgment.	Two single-item cue checks.

2.4 S7. Open-ended reframing rubric

Supplementary Table S6 defines the 0–3 reframing-quality scores. Score 3 requires a fact-inference distinction, an alternative explanation, and a balanced conclusion; it does not refer simply to a more positive statement.

Table S6. Open-ended reframing-quality coding rubric.

Score	Coding rule
0	No alternative thought, no response, or simple repetition of the original negative thought.
1	Simple reassurance or a general positive statement without an alternative explanation or evidential basis.
2	An alternative explanation is offered, but evidence testing is limited and the distinction between fact and inference is incomplete.
3	The response distinguishes facts from inferences, offers an alternative explanation, and reaches an evidence-based balanced conclusion.

Two independent coders rated all responses while blind to condition. Forty responses were used for training and revision of the coding manual before formal coding. Weighted kappa was .78 and ICC(2,1) was .82. Coding disagreements were resolved through discussion and the consensus score was used for analysis.
